# An Autonomous Robotized Cell for Polymer Foaming

**DOI:** 10.1021/acsomega.6c07764

**Published:** 2026-10-01

**Authors:** Valerio Loianno, Riccardo Caccavale, Massimiliano Maria Villone, Alberto Finzi, Ernesto Di Maio

**Affiliations:** † Department of Chemical, Materials and Production Engineering, 9307 University of Naples Federico II, P.le V. Tecchio 80, Naples 80125, Italy; ‡ Department of Electrical Engineering and Information Technology, University of Naples Federico II, Via Claudio 21, Naples 80125, Italy

## Abstract

This work represents the first step toward the implementation of a self-driving laboratory for polymer foaming. We exploit robotics, process control, and simple optical measurements to conduct the foaming process and analyze the quality of the produced foam. The experiments are autonomously driven by a computer, and the product analysis allows the adaptive tuning of the foaming process. A robotic manipulator handles the polymer samples, and the control system is responsible for the experiment execution, supervision, and evaluation. We choose poly-ε-caprolactone as a reference polymer material, and we propose a novel approach for the measurement of the foam density and the qualitative assessment of its morphology. To this aim, the system includes a customized testing platform for 3D scanning and RGB image acquisition of the foam top surface, which can be easily extended to the measurement of other material properties. We describe the automated experimental workflow and define the advantages and limitations of the density measurement and morphology analysis. Besides granting process optimization and high-throughput experimentation, the development of a self-driving laboratory for polymer foaming promotes safety, innovative teaching methods, and enhanced sharing of resources, materials, and equipment with research institutions and industry partners. The collection of big data will foster the implementation of an AI-driven experimental management.

## Graphical abstract

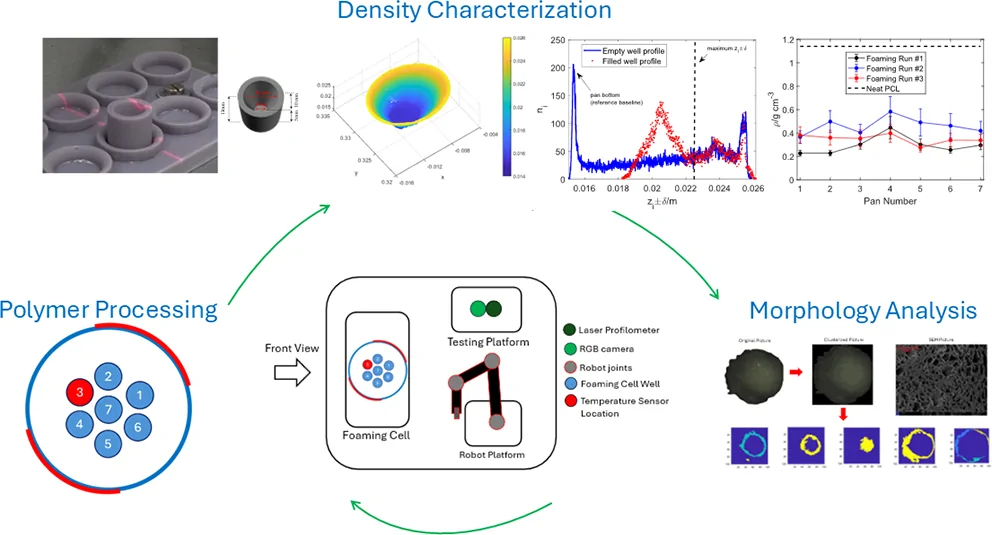

## 1 Introduction

Polymeric foams are of great interest for countless applications in both the technological-scientific and industrial fields (e.g., sports equipment, helmets, packaging, etc.).^1–3^ The diffusion of these materials is due to their versatility: by varying the density and morphology of the bubbles, as well as the chemical nature of the polymeric matrix, it is possible to modulate the properties, such as rigidity, resistance, thermal insulation, acoustic absorption, and impact protection. Every day, new foam-based products are placed on the market, which are innovative in terms of the polymeric matrix and/or the properties of the foam.^4^ Recently, the importance of the environmental impact of foams has also emerged, and future efforts must focus on the development of new sustainable materials and the definition of the related foaming process parameters to enable valuable collection and prevent dispersion in the environment.^1^

The process of giving the polymer a foamed structure is called “foaming”.^5^ Among the foaming processes, the most widely used and industrially important is gas foaming. A gas, e.g. , carbon dioxide, nitrogen, light hydrocarbons, or a mixture of gases acts as a blowing agent: it is first solubilized into the polymer at high pressure and, subsequently, following a sudden release of pressure, escapes the solution to form bubbles in the polymeric matrix.^6,7^ Over the past three decades, many authors have thoroughly investigated the foaming of standard polymers (e.g., polyethylene,^8^ polypropylene,^9^ polystyrene,^10^ poly­(methyl methacrylate),^11^ and polycarbonate^12^) with environmentally benign gases, such as carbon dioxide and/or nitrogen. Some studies have been carried out on the production of foams from biodegradable polymeric matrices, such as polycaprolactone and polylactic acid, which are of great economic interest.^13–15^ In particular, detailed analyses of the foaming processes have been performed in terms of the effects of the process parameters on the final foam structure.^16^ Typically, in the laboratory practice, a foaming experiment lasts for hours and involves long waiting periods for temperature equilibration and blowing agent sorption, which can be tedious (unless new, more involving procedures are put in place). Generally, a trial and error approach is followed to optimize the values of the several variables affecting the process.^17^ Establishing the so-called foam processing window, i.e., the correlation between process variables and the resulting foam features to find the optimal foaming conditions, may require week-long campaigns.^18^ Process variables are not limited to the temperature and pressure of the gas phase, but also extend to the composition and nature of the blowing gases.^19^ The great number of variables hinders a complete understanding of the foam processing window within reasonable time frames. Human intervention is limited to opening/closing the pressure vessel and changing the set point values of the process variables. Process control activities are simple but often dangerous since high pressure and temperature are needed to obtain the final product. From this perspective, a self-driving laboratory (SDL) can meet higher standards of safety measures.^20–23^ Recent reviews on this topic outline the evolution of SDLs. The first laboratory automation and combinatorial chemistry appeared in the 1980s–1990s, when machines executed repetitive tasks, but humans still designed the experiments. During the 2000s–2010s, improved robotics, flow chemistry, online analysis, and machine-learning optimization enabled platforms to select experiments using previous results. Since the 2020s, SDLs have developed from specialized systems into increasingly autonomous, modular, and multipurpose platforms capable of faster discovery, reduced reagent consumption, and richer, more reproducible data generation. The results obtained so far confirm that SDLs are not replacements for scientists, but scientific collaborators that amplify human capabilities.^24–28^ Their greatest prospective benefit is the creation of human–AI–robot teams capable of conducting experiments with a combination of speed, complexity, accuracy, and data fidelity that humans or conventional high-throughput systems cannot achieve alone. However, these benefits depend on overcoming limitations in hardware flexibility, scalability, interoperability, data standards, safety, and human oversight. Therefore, the strongest benefits are presently demonstrated in specialized applications, while globally networked and broadly general-purpose SDLs remain a future objective.

In this work, we propose an autonomous platform for polymer foaming based on a collaborative robotic manipulator which displaces the samples into/from a foaming vessel and then analyzes their properties by means of specific measurement tools. With this self-driving setup, larger ranges of values of the process variables, e.g., temperature, pressure, composition, and type of blowing agents, can be explored.^29,30^ The deployment of robotics and AI methods to manage flexibly and autonomously self-driving experiments in material research laboratories is considered very promising and appealing.^20–28,31–33^ For example, a very recent work describes the use of a mobile robot equipped with a manipulator for the management and conduction of a chemical reaction, with AI intervention for the identification of the optimal operating conditions, which is justified by the number of available catalysts and experiments to be carried out (about 1000 in 10 days).^32^ Another interesting approach was proposed by MacLeod and co-workers.^31^ Remotely guided experiments have also been proposed by Cloud Chemistry projects;^33^ however, in that case, the experimental activities were carried out by operators in the laboratory, while autonomous robotic platforms were outlined as possible future scenarios. Inspired by this last work, a simple robotic platform to perform a plastic foaming experiment remotely was recently proposed by our group.^33^ We now aim to extend this approach for the development of flexible and robust experimental management through a self-driving platform for polymer foaming. We describe the architecture governing the setup and the system we implemented for the foam volume/density measurement and qualitative morphology assessment.

In the literature, several standard methods are used to measure the volume of plastics and of polymer foams.^17^ Among them, the ASTM standard D792 is a fluid displacement method, while the ASTM standard D3574 refers to the measurement of the foam dimensions with a caliper and height gauge, provided the shape and geometry of the sample are well-defined.^34,35^ Noteworthy, cellular plastics may have open or closed cavities, and true and apparent densities can be defined. The former refers to the volume effectively occupied by the material, while the latter refers to the total specimen volume, including the volume of the void cavities. The ASTM standard D1622 defines the terminology and procedure to measure the apparent density based on geometrical measurements with a caliper. The true density of the polymer foam can be measured instead with a pycnometer that uses two calibrated volumes separated by a valve and helium as the gas to be expanded between them. This method works well for specimens with nonregular shapes, but the solubility of the gas must be negligible in the polymer dense skin.^36^ Alternative additional methods use a capacitance probe to measure dielectric constant changes related to foam density;^37,38^ THz pulses to map the internal refractive index of the foam, which correlates to the density distribution;^39^ high-resolution imaging based on X-ray CT/Micro-CT to reconstruct foam microstructure and infer density distribution;^40^ cryogenic scanning electron microscopy^41^ and x-ray radioscopy;^42^ and optical imaging.^43^ The methods capable of elucidating the internal structure of the foam can also be used to describe the shape and distribution of bubbles within the material, i.e., the morphology of the foam. As outlined by Krishnan et al., each method has advantages and drawbacks in terms of depth of penetration, in situ measurements, scan speed, destructive effects such as polymer degradation due to radiation, spatial and temporal resolution, cost, and additional instrumentation required to conduct the experiment, such as a vacuum setup.^17^ We define in this work two methods that can be easily adapted to the autonomous polymer foaming platform to assess the foam volume and morphology. The former is quantitative, while the latter is qualitative. The advantages and drawbacks are outlined.

## 2 Materials and Methods

Poly­(ε-caprolactone) or PCL in the following type CAPA 6800 purchased from Perstorp (Cardiff by the Sea, CA, USA) was adopted as a model polymer. This material has a glass transition temperature of −60 °C and a melting temperature of 60 °C. The density of the as-received material is 1.140 g cm^–3^ at 1 bar and 25 °C, with granules having an average diameter of approximately 3 mm. Technical grade CO_2_ was supplied by SOL S.p.A. (Monza, Italy).

### 2.1 Architectural Overview, Supervision, and Control

We designed a robotized platform that performs and evaluates the foaming tasks autonomously. Therefore, we conceived a software architecture (called the executive system in the following) based on the Robot Operating System environment (ROS), which controls the hardware components, i.e., the robot, the foaming autoclave, and the testing platform (see Figure 1). Once a foaming experiment has been performed, the executive system autonomously decides how to change the operating conditions to optimize the foam properties (the density and morphology).

**Figure 1:**
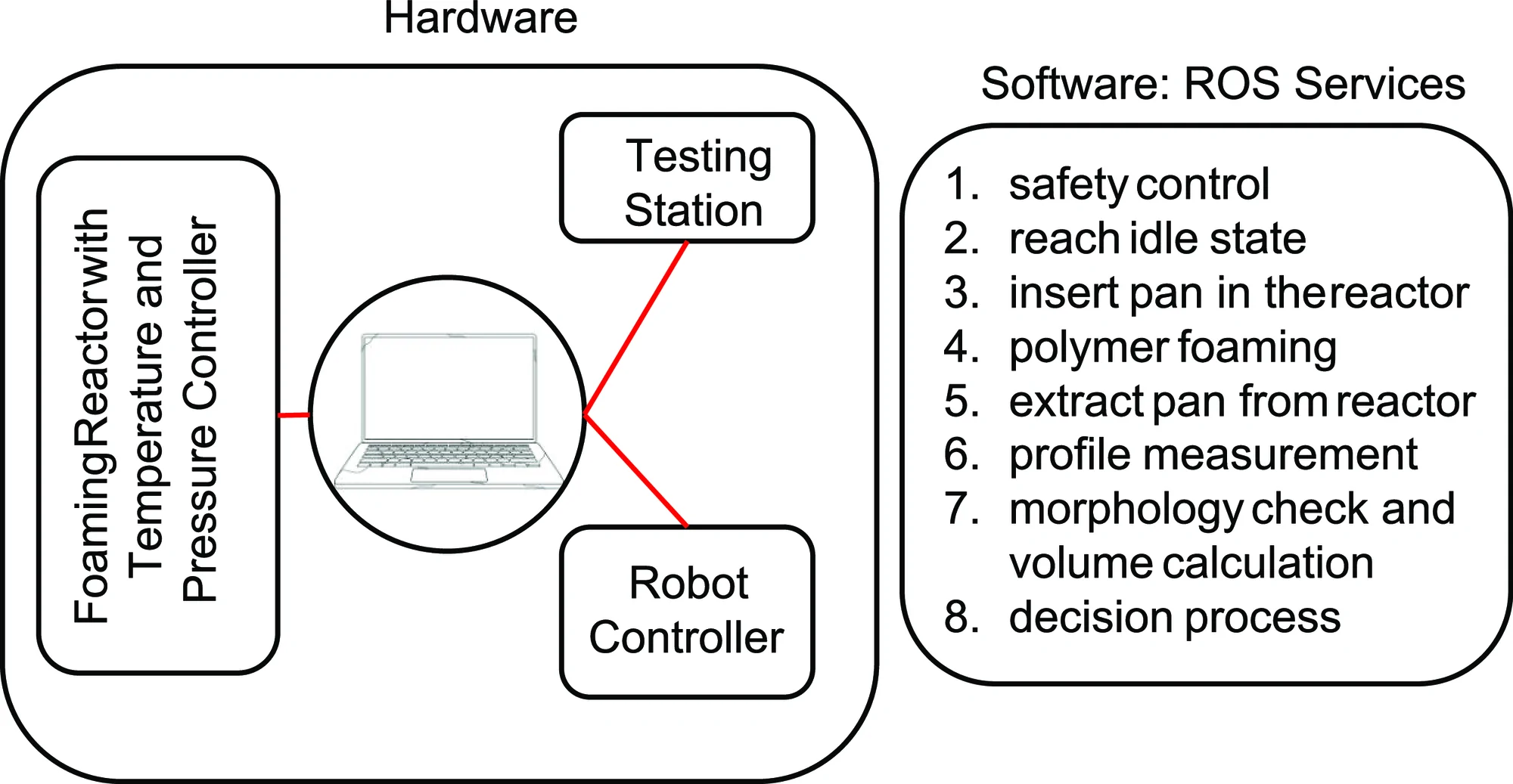
Schematic representation of the SDL platform. Hardware components include the robot manipulator, the foaming cell, and the density/morphology testing station. Separate algorithms communicate with each other to execute the experiment while ensuring safety.

The activities defining the foaming experiment are ROS services operated in series by the executive system.^11–13^ In Figure 1, the list of activities is presented following the current order of execution. New activities are easily introduced if needed (e.g., additional property measurements). Different error scenarios have been considered, and safety protocols autonomously implemented by the executive system have been introduced. Nevertheless, the platform grants intervention by remote users to check for errors and anomalies during execution. The deployed executive system potentially enables a local operator to physically interact with the collaborative robot to teach new tasks, but this feature is currently not being exploited. A record of the executed tasks and encountered errors is implemented to make the platform completely autonomous.

### 2.2 Setup of the Robotized Foaming Cell

The design of the proposed foaming cell is depicted in Figure 2. The cell is composed of three main elements: a manipulating robot, a foaming vessel, and a testing platform for analyzing the foam properties. The cell configuration is like that proposed in ref 44: the robotic arm is fixed with respect to the other instruments (vessel, sensors, polymer dispenser, etc.) so that the positions for pick-and-place operations can be calibrated.

**Figure 2:**
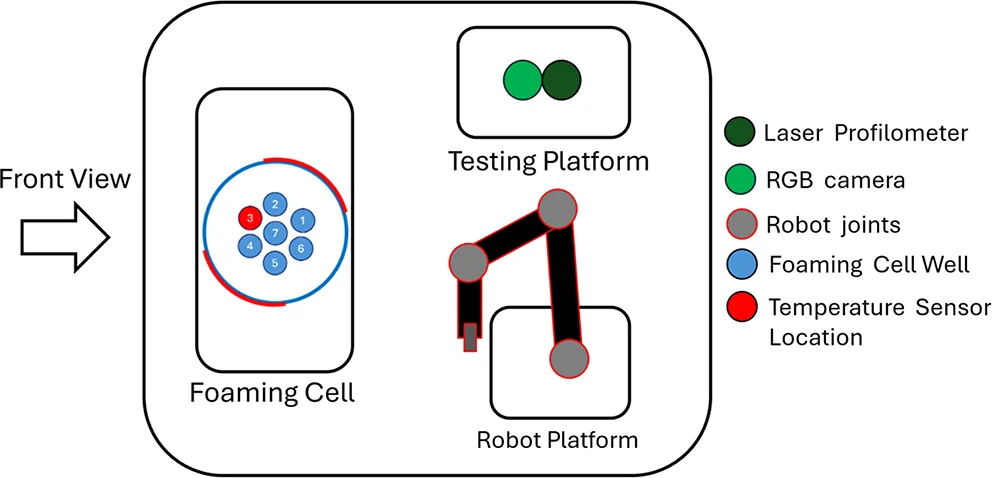
Schematic diagram of the setup components. Relative positions reproduce the real setup configuration. The foaming cell includes seven cylindrical wells hosting the sample pans and one point for the temperature measurement (well #3). A picture of the setup from the front view is visible in Figure 3.

The robot is a DOBOT MG400 lightweight 4-axis collaborative manipulator with 750 g of payload, a workspace of 440 mm, and a nominal repeatability of ±0.05 mm. The robot features a sucking terminal device to pick, carry, and place the pans containing the polymeric samples during the experiments. Pick-and-place operations are monitored by checking the positions of the robot head with respect to the ones previously calibrated. The operation fails whenever the executive system notices a mismatch between the final position attained by the robot during one movement step and the calibrated one, or if the robot itself shows a specific error (e.g., motor break, communication lost).

The foaming device, a pressurized vessel with pressure and temperature control,^9,10^ is designed to be compatible with the capabilities of the robot and autonomous control. It is positioned frontally with respect to the central robot, and the closure mechanism is based on a hydraulic press, leaving enough space for the robot to move between the plates and insert the polymeric samples into specific slots at the lower end of the device, as depicted in Figures 2 and 3. The foaming process is controlled with a 3850T programmable logic controller from Gefran S.p.A. (Brescia, Italy). The reactor closing/opening is activated with a bench hydraulic press type VLP106PAT1U of 10 tons from Enerpac S.p.A. (Milan, Italy). The system is heated with eight heating cartridges inserted in the stainless-steel walls of the foaming reactor. Four of them also include feedback temperature sensors (type K thermocouples). The system is cooled by heat exchange with the surrounding atmosphere. In Figure S1, the temperature and pressure evolution with time during independent heating/cooling scans is presented. The time needed to reach a steady state temperature of 70 °C during heating is equal to 2500 s. During cooling, 3600 s are sufficient to decrease the temperature down to 45 °C approximately, guaranteeing effective foaming of the sample. The volume measurement is conducted with an optical sensor type OXP200-R05C.004 from Baumer Italia S.p.A. (Milan, Italy). The reported distance resolution (*z*-axis) is 8–15 μm with a repeat accuracy of 4 μm. The field of view is 48–72 mm, and the resolution over the *y*-axis is 80–120 μm (see the “testing 1” block in Figure 2). A stepper motor governs the linear motion of the profilometer so that the resolution along the *x*-axis is equal to 20 μm. Pictures of the foamed samples are taken with a 2.0-megapixel camera type ELP-USBFHD01M-SFV (see the “testing 2” block in Figure 2).

**Figure 3:**
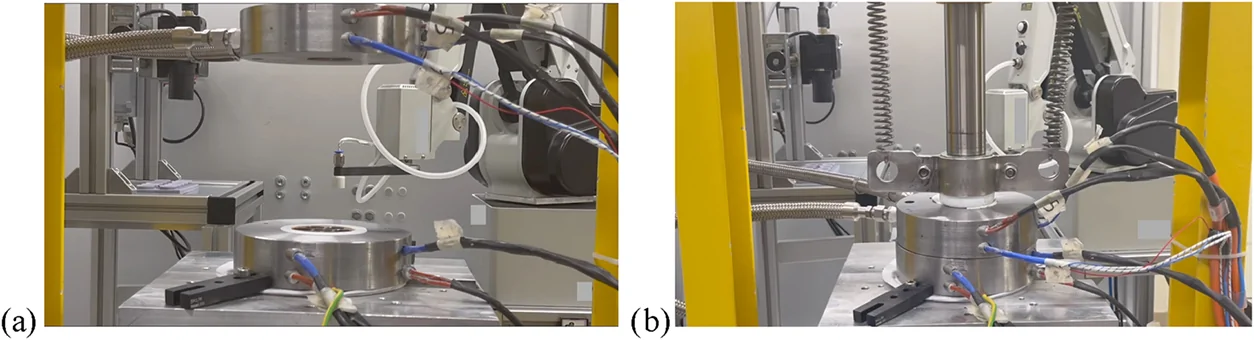
Front view picture of the setup, with the autoclave in the foreground. (a) reactor open; (b) reactor closed. The testing station and the robot are in the background.

In Figure 3a, we show the front view of the setup with the reactor closed during the foaming experiment. The evaluation of the outcomes of a foaming test is performed by measuring the density and assessing the morphology of the produced foams through the laser scanner and the camera positioned on the testing platform. In Figure 4, a picture of the instruments is presented.

**Figure 4:**
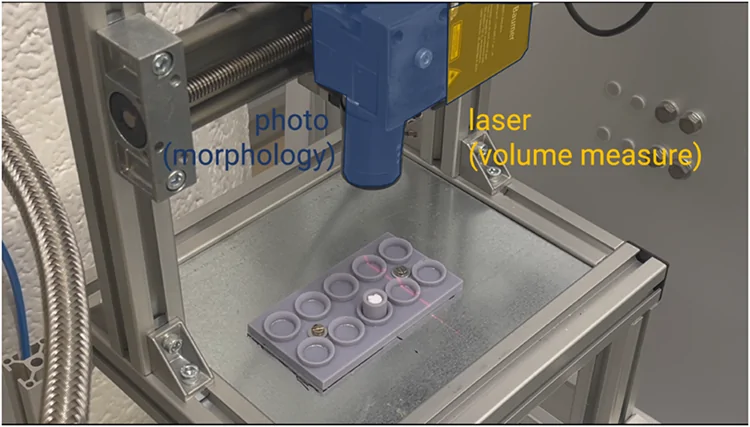
Picture of the testing platform, with the camera for the morphology assessment and the laser for the density measurement.

The shape of the pan in which the sample is confined and foamed was precisely designed to optimize the optical profile measurement. Specifically, the optical sensor OXP200-R05C.004 from Baumer measures a distance profile from the sensor by detecting the beam reflected from the scanned object. The reflected beam is not perpendicular to the plane upon which the object is located, and some parts can be shadowed during the scanning process. We initially built cylindrical pans made of polytetrafluoroethylene and observed that their internal walls acted as obstacles for light detection. To overcome this issue, we resorted to a 3D resin printer from Elegoo EU to produce the ad hoc sample pans. The height and outer diameter of the circular pan were equal to 13 mm. The internal volume where the polymer beads are foamed has a depth of 10 mm and a conical shape. The internal diameter varies in the range of 10–4 mm from top to bottom, respectively, of the internal volume (see also Figure 5a).

**Figure 5:**
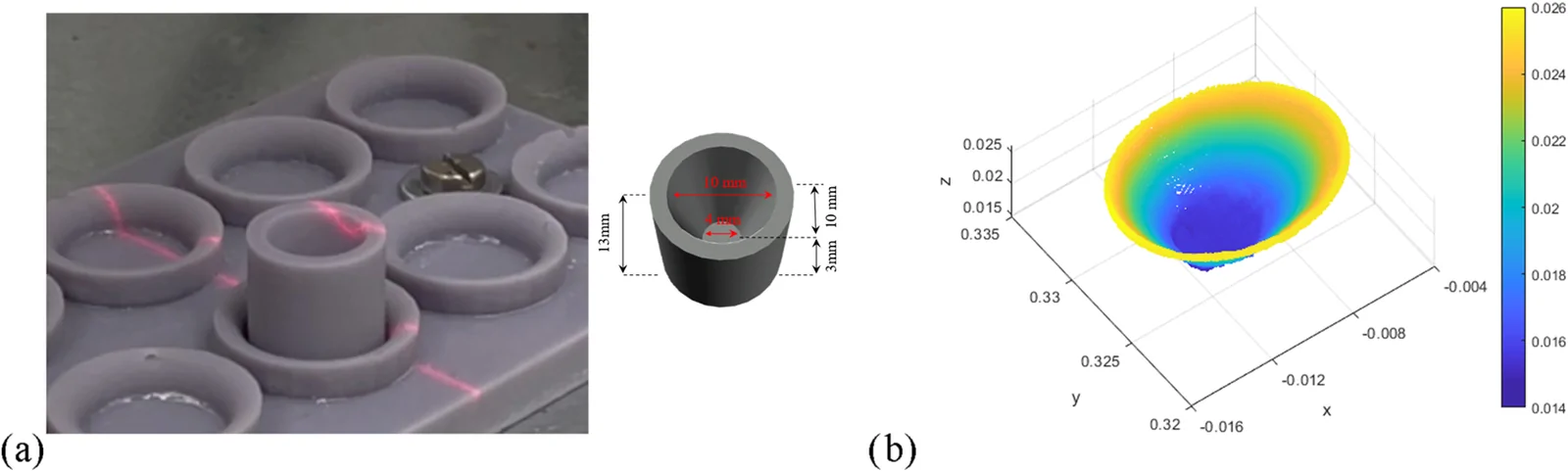
(a) Details of volume measurement with the empty sample holder. (b) Cloud point depicting the internal surface of the pan.

### 2.3 Analysis of the Results

Different methodologies are considered for the design and interpretation of experiments. The positive/negative outcome of the foaming process is assessed by measuring the density of the produced foams and the quality of the foam morphology. The latter is generally evaluated in terms of the number and dimensional distribution of the bubbles and their primary mechanical characteristics (elastic modulus and strength). As a starting point, we estimated the density of the foam from its volume and weight. Foaming experiments are validated by evaluating the density of the foamed samples after combining a mass measurement, performed with a classical analytical microbalance, and a volume measurement, performed with a height profilometer. The stiffness and strength of the foam can also be measured using a load cell mounted on the robotic arm, which is not included in the present setup. Regarding the morphology measurement, we deploy a simple optical system combined with image recognition routines to measure the degree of bubble segregation based on the brightness and color of the upper surface of the foamed samples. This protocol provides qualitative information about the morphology of the foamed sample. The selected features will be used to define quantitative metrics for process tuning, enabling the identification of effective foaming conditions for a given polymer type and a target foam. Three process variables mainly affect the density and cellular morphology of the polymer foam: (1) the foaming temperature, (2) the blowing agent type and concentration, and (3) the pressure release rate.

## 3 Results and Discussion

The volume measurement follows the approach proposed by Sun et al.^45^ The authors recently proposed a depth-camera-based measurement methodology to determine the volume of the bulk stack in the transfer process of liquor-making production. In that case, the depth sensor had an optical resolution/uncertainty on the order of millimeters or even tens of millimeters with a measured volume on the order of hundreds of dm^3^. In this work, we resort to a profilometer capable of determining volumes smaller than 1 cm^3^. Figure 5b shows a typical cloud point profile of an empty pan measured with our sensor.

The volume underneath the surface profile in Figure 5 can then be measured as described below. The cloud point profile is divided by *z*_*i*_ values grouped in specific height ranges equal to *z*_*i*_ ± δ, where δ is equal to 1 μm. For each group, an occurrence number *n*_*i*_ is determined. The occurrence number is defined as the number of points included in the height range. The 3D cloud point is converted with this approach into a 2D diagram of *z*_*i*_ ± δ vs number of occurrences *n*_*i*_. The distance between two adjacent points *z*_*i*_ and *z*_*i+1*_ along the *x*-axis is defined by the speed of the brushless motor and the thread pitch of the screw along the *x*-axis. The distance between two adjacent points along the *y*-axis is defined by the profilometer. Δ*y* and Δ*x* values are equal to 0.01 mm and 0.02 mm, respectively. Typical cloud point 2D diagrams for empty pans and pans filled with a polymer foam are reported in Figure S2. The following equation returns the volume as defined above:

1 $$V=\Delta x\cdot \Delta y\cdot \sum z_{i}$$

The volume of the foamed sample was measured by scanning the well profile without removing the sample from it at the end of the foaming process. In detail, the difference between the volumes of the empty and filled wells is calculated by resorting to eq 1. To visualize this measurement, the reader is referred to Figure 6. The two profiles correspond to the empty (blue line) and filled pans (red dots). The difference between them is exactly equal to the volume of the foamed samples. It is observed that when the foaming process is concluded, the specimen covers a specific area of the well, causing the disappearance of all *z*_*i*_ values smaller than a threshold *z*_i_* value. In Figure 6, we show that this value equals approximately 18 mm. For any *z*_*i*_ greater than the maximum value indicated with a dashed vertical line, the two profiles are coincident, confirming that it only refers to the well surface and, specifically, to the area uncovered by the foamed sample (see Figure 5). Then, the specimen profile is represented by the red dotted trace at *z*_*i*_ < *z*_*i*,max_. To minimize the measurement uncertainty, any *z*_*i*_ value greater than this value was discarded.

**Figure 6:**
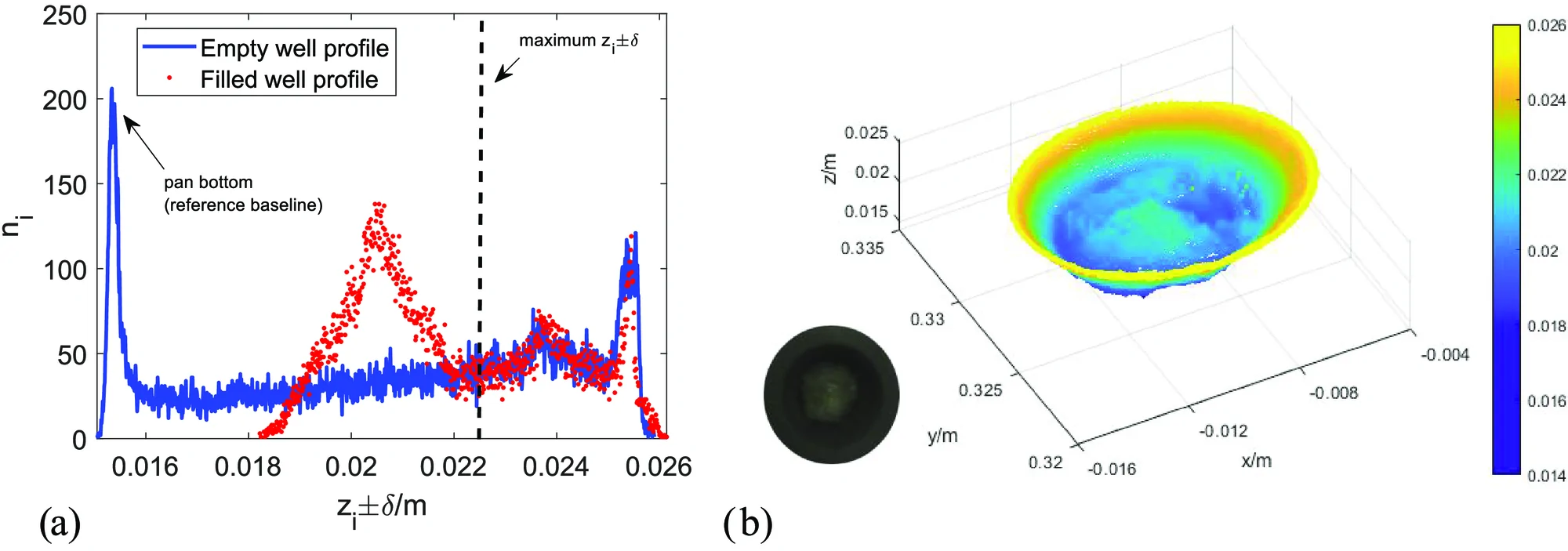
(a) Comparison between the profile scans of the empty well and the well filled with the foamed sample. (b) Cloud points of the foamed sample in (a) and a picture of the top surface.

The volume uncertainty is estimated to be equal to 0.01 cm^3^ at 95% confidence level by executing ten repeated profile scans on the same empty pan. Figure S2A reports this scan collection. Figure S2B shows the same data treatment for a pan including a foamed sample of PCL. We have estimated an uncertainty of 0.025 cm for the height measurement. A reference height value is generally chosen to fix the zero point and, provided that this value is kept invariant between consecutive measurements on the empty and filled pans, the measured volume is unaffected by it. To further validate the proposed approach for the volume measurement, we resorted to a standard method based on liquid water, as previously done in the literature.^46^ Liquid water (ρ = 1 g cm^–3^) is introduced within the well before the polymer specimen is loaded and after foaming it to fill the void volumes. The volume of the sample is measured from the difference between the two masses, knowing the density of the liquid. It is assumed that water absorption in the foamed polymer sample is negligible during the mass measurement. Also, we verified that the pan material is not soluble in water and that the pan mass and shape are invariant following this protocol. We have estimated an uncertainty of 0.01 cm^3^ when resorting to liquid water for the volume measurement, similar to the uncertainty of the optical method. The volumes of seven foamed specimens during the first foaming run (run #1) are measured using the standard method based on liquid water and the optical method proposed in this work, and the results are compared in Figure S3. The agreement between the two data sets confirms the soundness of the approach.

We have further analyzed the performance of the proposed method for the volume measurement by considering the effect of the polymer material, its refractive index, light transmittance, and surface reflectivity. First, we notice that the refractive index of polymers such as PCL, PLA, PP, PS, and PET is equal to ∼1.5 and decreases to ∼1.0 after foaming. Therefore, we do not consider in the following discussion the effect of the refractive index, which would be similar for all of these materials. Light transmittance depends on the refractive index of the material, the angle of the incident radiation, and the specimen thickness. Surface reflectivity depends on the angle of the incident radiation and the specimen thickness, besides the surface roughness. Therefore, we only focus on surface reflectivity and the parameters upon which it depends. We scanned the profile of a stainless-steel bearing having a polished and highly reflective surface. As shown in Figure S4, the incident light on the metal surface is not reflected to the detector, and a significant number of points are missing in the measured height profile. The top view clearly shows the missing points producing a white corona in Figure S4B. The small reflectance of polymeric materials hinders this phenomenon. We then scanned the height profile of two foams made of poly­(ethylene terephthalate) (PET) and polystyrene (PS), the latter used for heavy-duty protective cushioning. Neither the high surface roughness of the PET sample nor the smooth irregular surface of the PS sample affects the height measurement, as observed in Figures S5–S7. Provided the sample height value is within the optical field range of the sensor, the only relevant issue encountered during the height measurement of polymer foams is the presence of shadowed regions not accessible to the incident radiation and/or height changes with steep curvatures, which prevent the reflected beam from reaching the detector. Indeed, in this second case, the reflected beam may encounter an obstacle and cannot reach the detector. Figure S5D,F show missing points due to the reported issues. If just a small number of points are missing, the defective measurement can be easily corrected with a linear or quadratic interpolation. The irregular shape of the surface, the different surface roughness with respect to PCL samples, and the different thickness of the foam outer surface do not appreciably affect the height measurement.

In Figure 7, we report a schematic diagram of the wells’ positions in the reactor along with the volume measurement of three independent sets of foamed PCL samples. Well #3 is depicted in red to highlight the point where the temperature is measured. Also, the two red arcs indicate the position of the heating wires included in the reactor walls, with which we control the temperature of the cell. New PCL pellets were used during each independent run. We compare here the proposed measurement approach with the literature.^17^ First, the foamed sample strongly adheres to the sample pan and it is damaged when detached from it. The proposed method does not require any further manipulation of the foam after polymer processing, granting better results. The method also measures the apparent density of the polymer foam so that it is better suited to foams with closed cells. Optical imaging techniques are easy to use and fast, granting a higher yield. Clearly, the cost is also lower than that of imaging techniques, which require expensive facilities. The method can also be extended to sample holders with different shapes and dimensions and is very flexible in determining the volume of irregularly shaped polymer foams (see Figures S5–S7). Among the main drawbacks, we notice that this technique does not allow access to the 3D internal structure, and the true density of a polymer foam with open cavities cannot be resolved. The method was implemented so that the platform could work autonomously and produce large sets of data, which in the end will allow the inclusion of optimization processing algorithms or feed AI methods for the evaluation of the foaming process.

**Figure 7:**
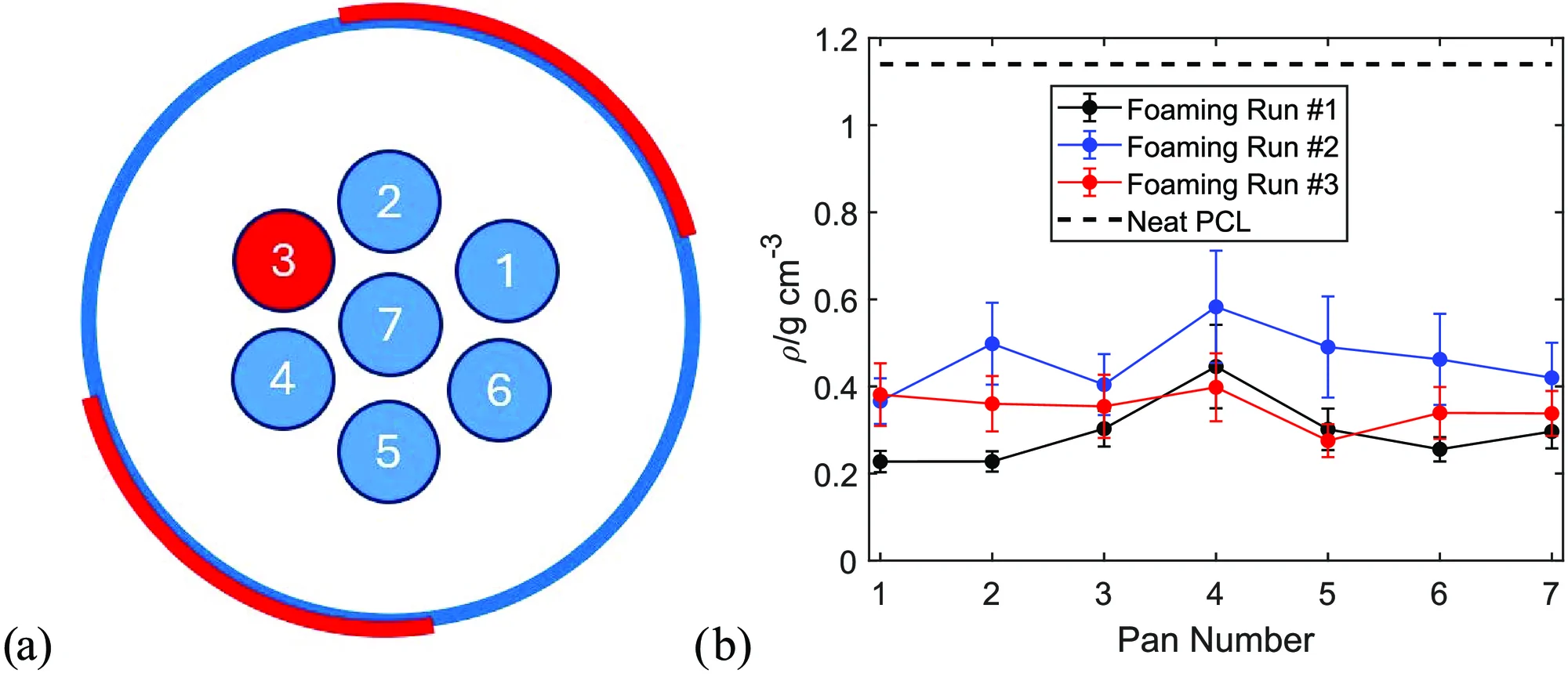
(a) Schematic diagram of the foaming reactor and wells’ positions (top view of the bottom part). Heating resistors fix the temperature at the red borders of the cell. The temperature and the pressure are measured at well #3. The dashed orange line depicts the theoretical plane of symmetry dividing the cell into two pieces with identical thermal profiles. (b) Density of the foamed PLC samples.

We further analyze the density values measured with the optical technique. We have complemented the experimental analysis with a theoretical description of the temperature profiles expected in the foaming cell with simulations in the COMSOL Multiphysics environment. The geometry is reported in Figure S9. We assume that heating cartridges operate as if the temperature were fixed on two opposite portions of the outer cell walls. The foaming cell has a cylindrical shape, and heat exchange with the surrounding atmosphere occurs via a boundary layer resistance characterized by a heat exchange coefficient equal to 15 W m^–2^ K^–1^. This value approximates the condition of mild convective flow in the ambient air due to air conditioning. We fix the ambient temperature to 25 °C in the simulations. The study includes two subsequent steps replicating the experimental heating-cooling cycle. Due to the presence of cold and hot spots on the outer surfaces of the cell, a condition of thermal equilibrium cannot be attained^47^ and, at steady state, a nonconstant temperature profile characterizes the foaming cell. The maximum temperature difference between wells is smaller than 1 °C during heating and 0.3 °C during cooling as estimated from the theoretical simulation. Based on the previous analysis, density variations between the three runs do not depend on temperature gradients in the system. Density differences are due to the specific foaming temperature/pressure window adopted: PCL is a semicrystalline polymer, and as such, during foaming, a competition may exist between the crystallization kinetics and bubble cell growth (melting temperature of pure PCL is 60°C). In addition, the high concentration of CO_2_ plasticizes the material, thus reducing the melting and crystallization temperatures with respect to the dry polymer.^18,48–51^ We complete the density analysis, taking advantage of literature data. The work by Rhee et al.^18^ studied the melting temperature depression of a PCL with a molecular weight analogous to the one tested in the present work. At 30 bar, the melting temperature is approximately 52 °C, and the crystallization temperature is approximately 37 °C. This interval gives the best balance between the attainment of sufficient mobility for cell nucleation and growth and sufficient crystalline structure or rapid recrystallization to stabilize the cells. The foaming temperature during run #1 was ∼47 °C (smaller than for runs 2 and 3, for which we registered ∼50°C). Clearly, the higher temperature in runs 2 and 3 was closer to the melting point and enhanced the collapse and coalescence of the formed bubbles, producing foams of greater density.

The final part of this study focuses on the definition of a qualitative approach to assess the foam morphology. The foaming process is considered successful when the porous structure is uniform in both pore size and spatial distribution.^52^ Typically, scanning electron microscopy (SEM) is used to reveal the foam morphology, while image analysis software enables the measurement of pore size distribution and bubble number density.^53,54^ At the current stage of robotic development, the automation of foam sample handling, blade cutting, and SEM analysis remains unfeasible. X-ray microtomography (micro-CT) represents a viable alternative that does not require sample cutting; nevertheless, it is still impractical in terms of sample handling and is associated with high equipment costs.^40,41^ In practice, even a qualitative assessment of foam morphology can be inferred from the external surface. Foams with a fine and uniform cellular structure typically exhibit a smooth, turgid, shiny, and locally convex surface, free of pits, wrinkles, or creases. Moreover, the presence of large bubbles caused by coalescence can be detected through visual inspection, and in digitally acquired images, this feature would appear with the color not being uniform and with shadowed regions. Based on these considerations, we implemented a macroscopic optical inspection setup and developed an algorithm to assess the foam morphology in terms of uniform and nonuniform distribution of pores. To validate this method, off-line SEM analyses were conducted on the samples, and the results were compared with those obtained from the automated optical assessment. We collected pictures of the upper surface of the foamed samples with a simple camera and defined a digital segmentation protocol to identify uniformly (good) and nonuniformly (bad) distributed pores within the prepared samples. This analysis is qualitative by nature since we cannot inspect the internal structure of the foam. In Figure 8, a foamed sample with a uniform morphology is presented. The original image shows a unique, rounded dome spanning a large part of the picture. The image segmentation was conducted using the *k-means* method: pixels with similar colors were initially grouped into clusters. The total number of clusters was fixed at five, following the elbow optimization method. This method considers that an increase in the number of clusters beyond the optimal value does not improve the final segmentation and is based on the total squared distance between each data point and the centroid of the cluster to which it belongs. Once the number of clusters has been defined, pixels are further grouped in each cluster based on an 8-numbered two-dimensional connectivity method, i.e., pixels are connected if their edges touch. As reported in the figure, this second step is useful to highlight how pixels are distributed in each cluster throughout the image. When the porous structure is uniformly distributed, pixels are uniformly grouped within each cluster, and a central inner circle enclosed in coronas with larger radii is recognized. The SEM micrograph is reported in the same figure to depict the uniformly distributed porous structure of the foam.

**Figure 8:**
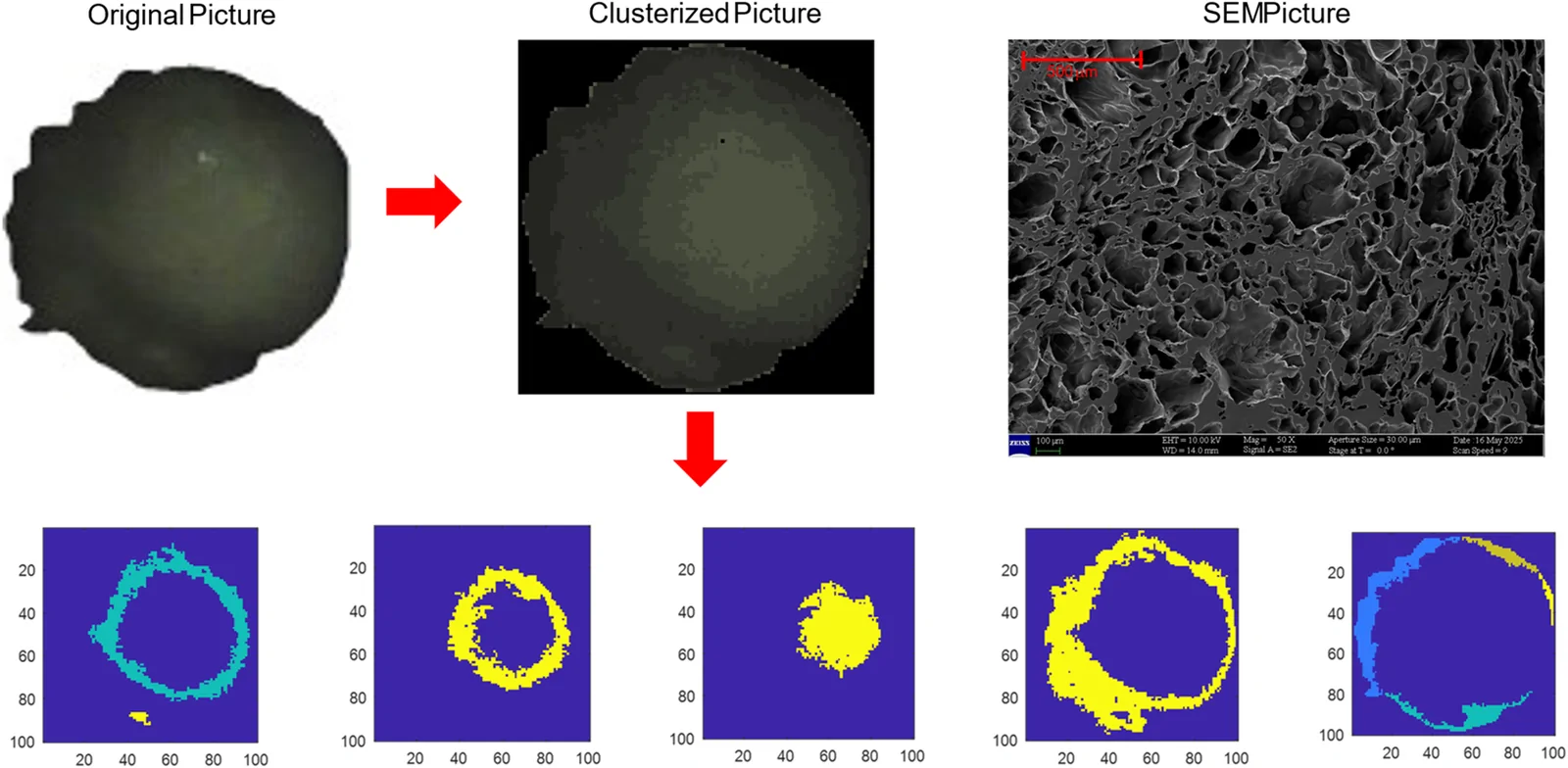
Qualitative analysis of foam morphology: random distribution of pores with a homogenous structure. The original picture is segmented into five clusters separated from each other in the five subplots. Each cluster is defined by islands of interconnected pixels shown with different colors on the blue background. SEM images of the same sample are reported for comparison.

In Figure 9, we report the case of a foamed sample with nonuniformly distributed pore sizes. We recognize that, by following the same image segmentation protocol adopted in Figure 8, pixels in each cluster are grouped and distributed in the form of asymmetric coronas, including holes that identify different porous structures. The number of subgroups per cluster is defined by different colors (yellow to light blue) and does not change with respect to the case of samples with a uniformly distributed pore size. Therefore, the shape of the subgroups identifies the quality of the foam based on the proposed segmentation method. We are aware that this approach cannot compete with the quantitative microstructural analysis performed with micro-CT and scanning electron microscopy. At present, the incorporation of these techniques into an SDL platform is limited by cost and complexity. Therefore, one of the goals of the present work has been to provide a viable solution for the determination of the quality of the foam morphology.

**Figure 9:**
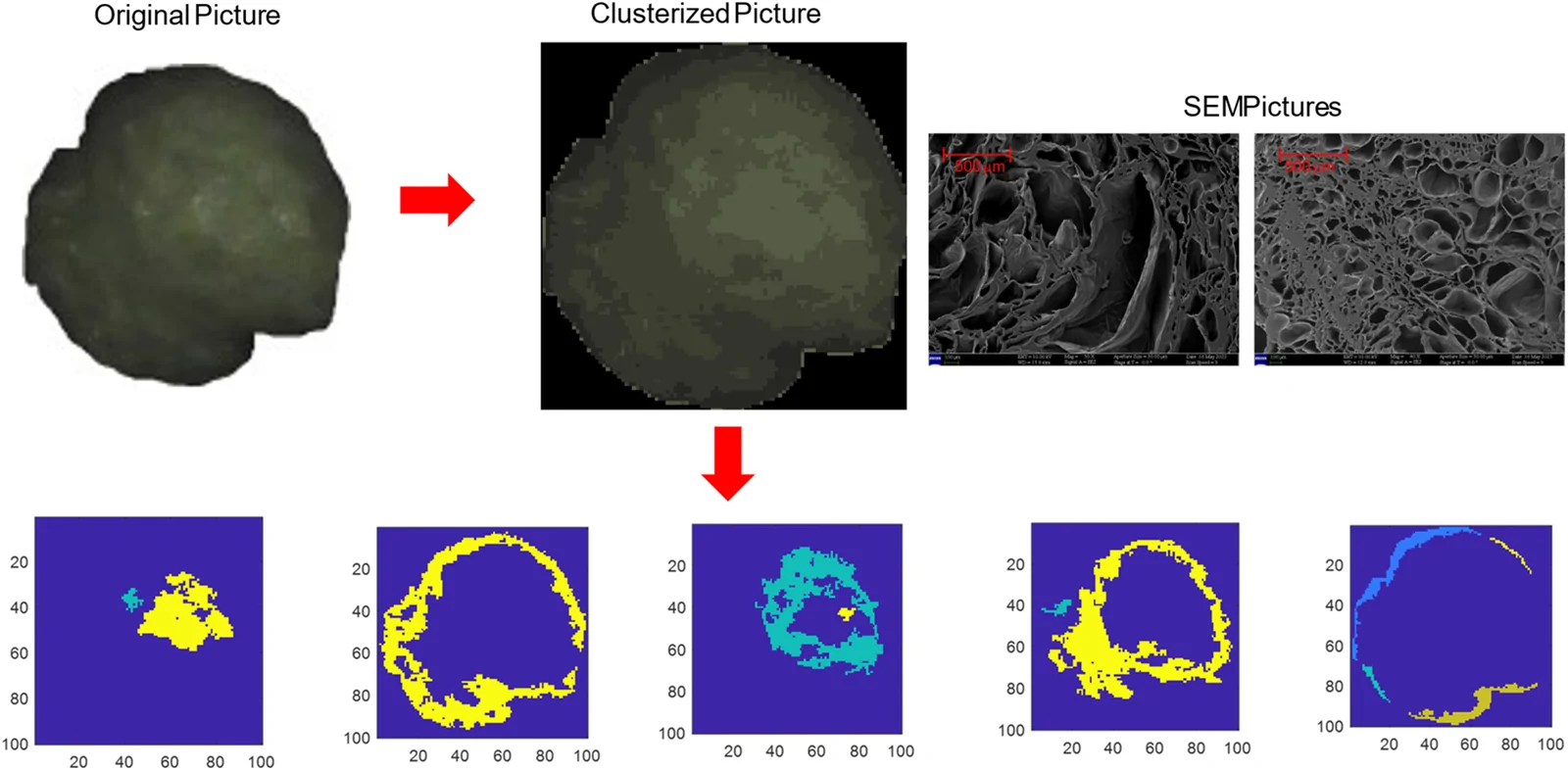
Qualitative analysis of foam morphology: random distribution of pores with nonhomogeneous structure. The original picture is segmented into five clusters separated from each other in the five subplots. Each cluster is defined by islands of interconnected pixels shown with different colors on the blue background. SEM images of the same sample are reported for comparison.

Work is in progress to operate the entire system with AI-based methods for an autonomous experiment configuration and adaptive tuning of the foaming conditions. These methods will be trained by using experimentally acquired data and the approaches proposed in this work.

## 4 Conclusions

A self-driving platform was implemented for polymer foaming. The method allows the autonomous execution of the process and the characterization of the final foamed sample. Specifically, the density was quantitatively measured from the ratio of the sample’s mass and volume. An optical method based on a height profilometer was defined and validated to measure the volume of the foam. A qualitative method was proposed for the assessment of the foam morphology. A simple camera was used to depict the upper part of the foamed sample, and its morphology was analyzed with an unsupervised machine learning algorithm to group unlabeled data into distinct clusters based on their similarities. Poly­(ε-caprolactone) was chosen as a model material to test the autonomous platform. The volume uncertainty was estimated to be 0.01 cm^3^, and foamed samples with densities ranging from 0.2 to 0.6 g cm^–3^ were obtained. The morphology was assessed in terms of uniformly and nonuniformly distributed pore sizes. The processing/measuring platform can be used either to train machine learning algorithms for the assessment of the foam properties or to optimize the foaming process parameters. The proposed approach can be easily extended with additional characterization units, such as for example the measurement of the foam mechanical properties.

## Supplementary Material



## Notes

Additional information on the thermal and pressure profiles, the repeatability of the height profiles, the uncertainty of the volume measured by comparison with a standard approach, and supplementary information regarding laser scanner performances. (PDF)
