# Supplementary material for: An Autonomous Robotized Cell for Polymer Foaming
Source: ACS Omega. 2026 Oct 1;11(40):61887–96. doi: 10.1021/acsomega.6c07764 (PMC13670802; doi:10.1021/acsomega.6c07764)
Supplement: Supplementary file 1 [file ao6c07764_si_001.pdf]

An Autonomous Robotized Cell for Polymer Foaming

Supporting Information

Valerio Loianno[a*], Riccardo Caccavale[b], Massimiliano Maria Villone[a], Alberto Finzi[b], Ernesto Di Maio[a]

[a]Department of Chemical, Materials and Production Engineering, University of Naples Federico II, P.le V. Tecchio 80, Naples, 80125, Italy

[b]Department of Electrical Engineering and Information Technology, University of Naples Federico II, Via Claudio 21, Naples, 80125, Italy

*corresponding author valerio loianno valerio.loianno@unina.it

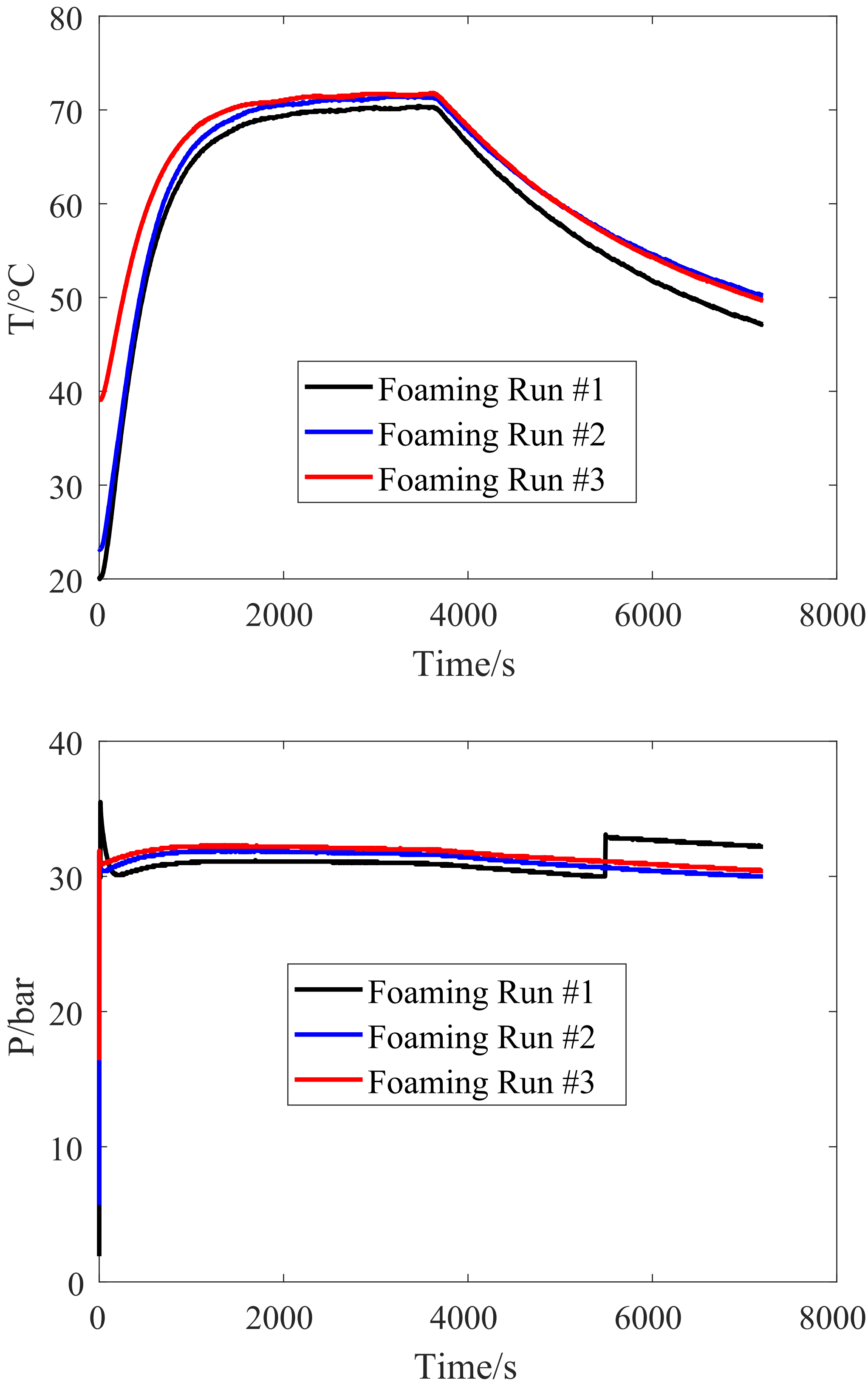

Figure S1. Temperature evolution with time during independent foaming scans

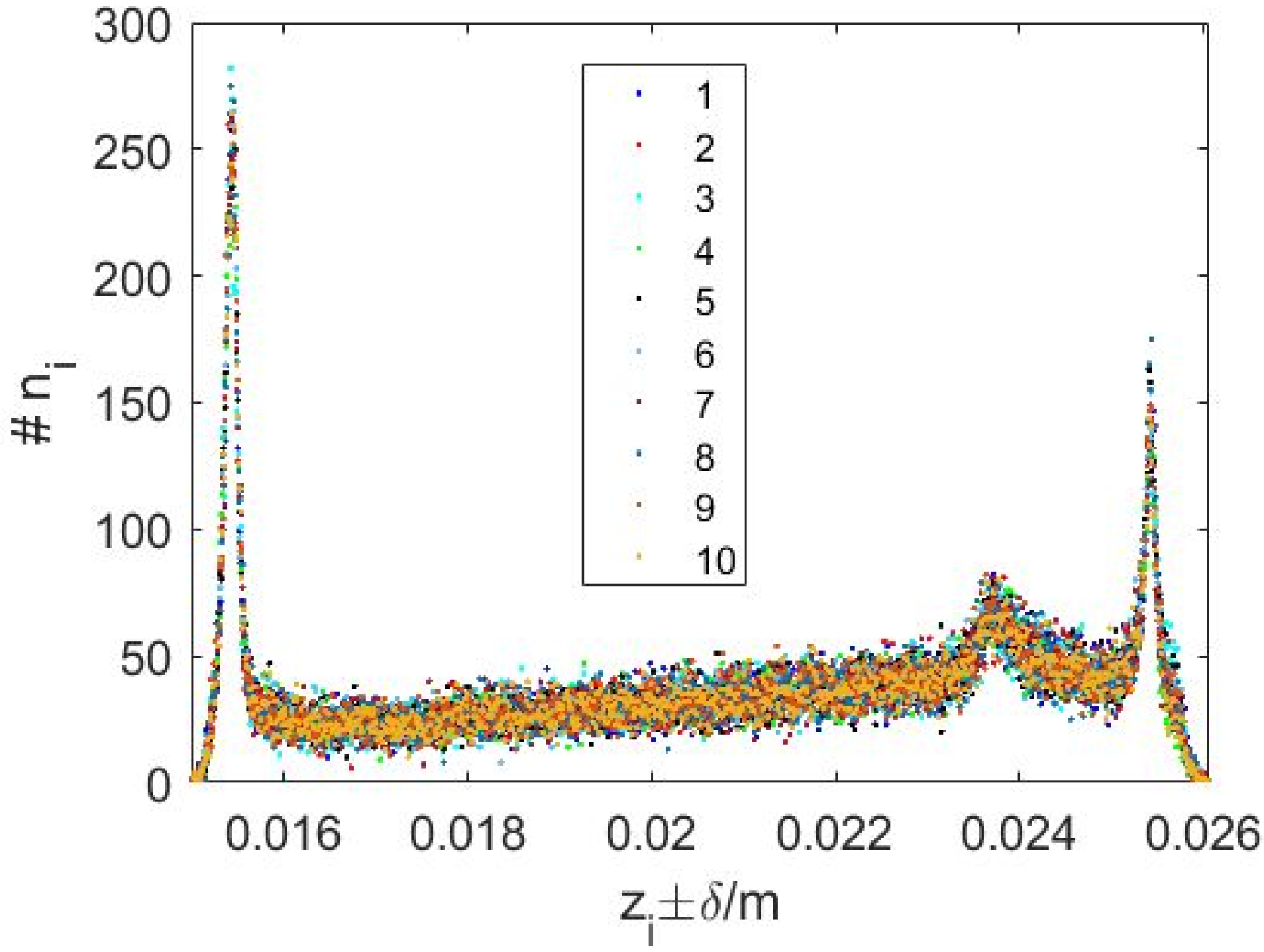

a)

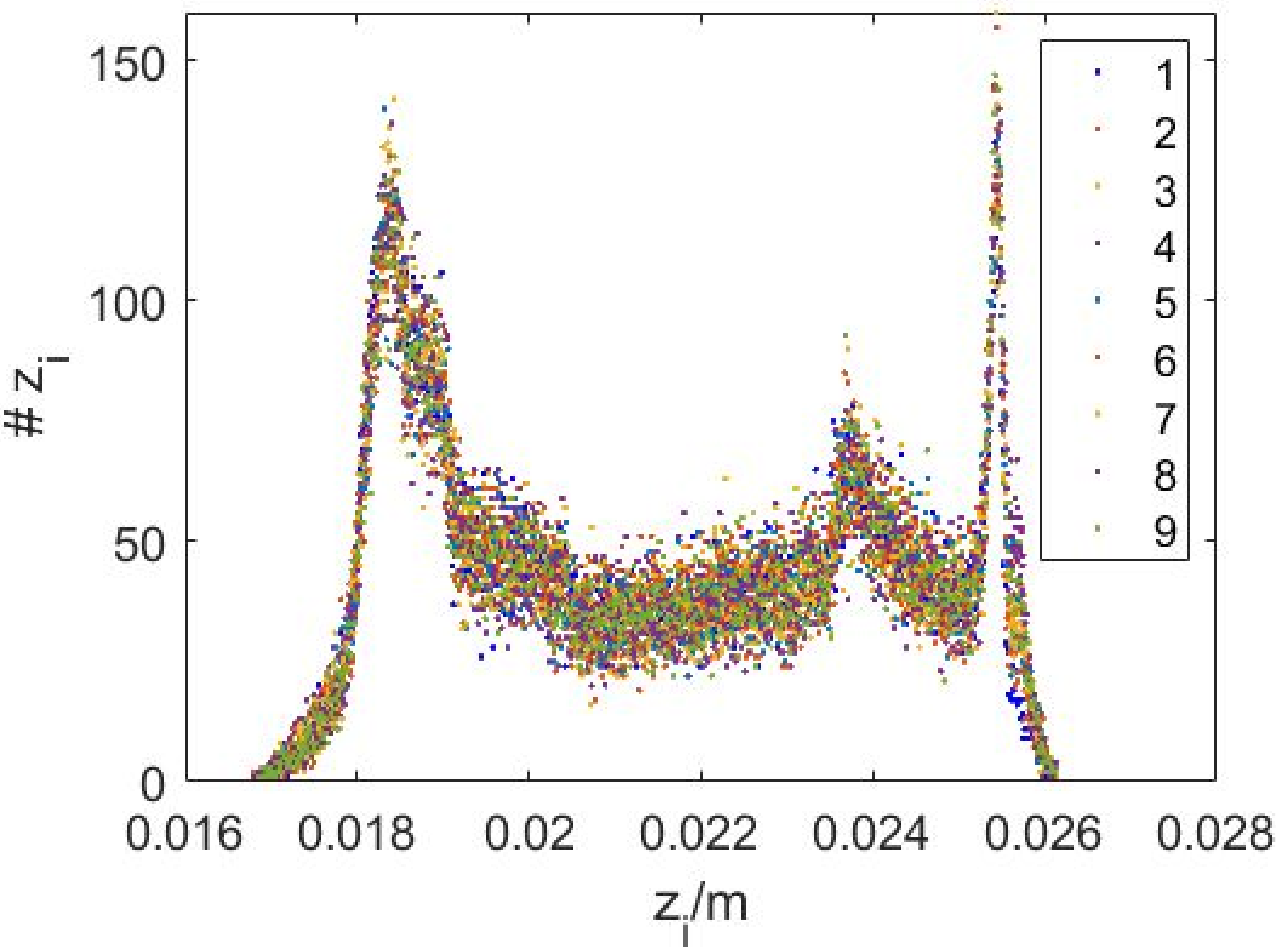

b)

Figure S2. 2D cloudpoint profile of: (a) empty pan; (b) pan filled with foamed PCL. Repeatability of the measurement from multiple subsequent scans. The height uncertainty estimated from this analysis is equal to ±250 μm.

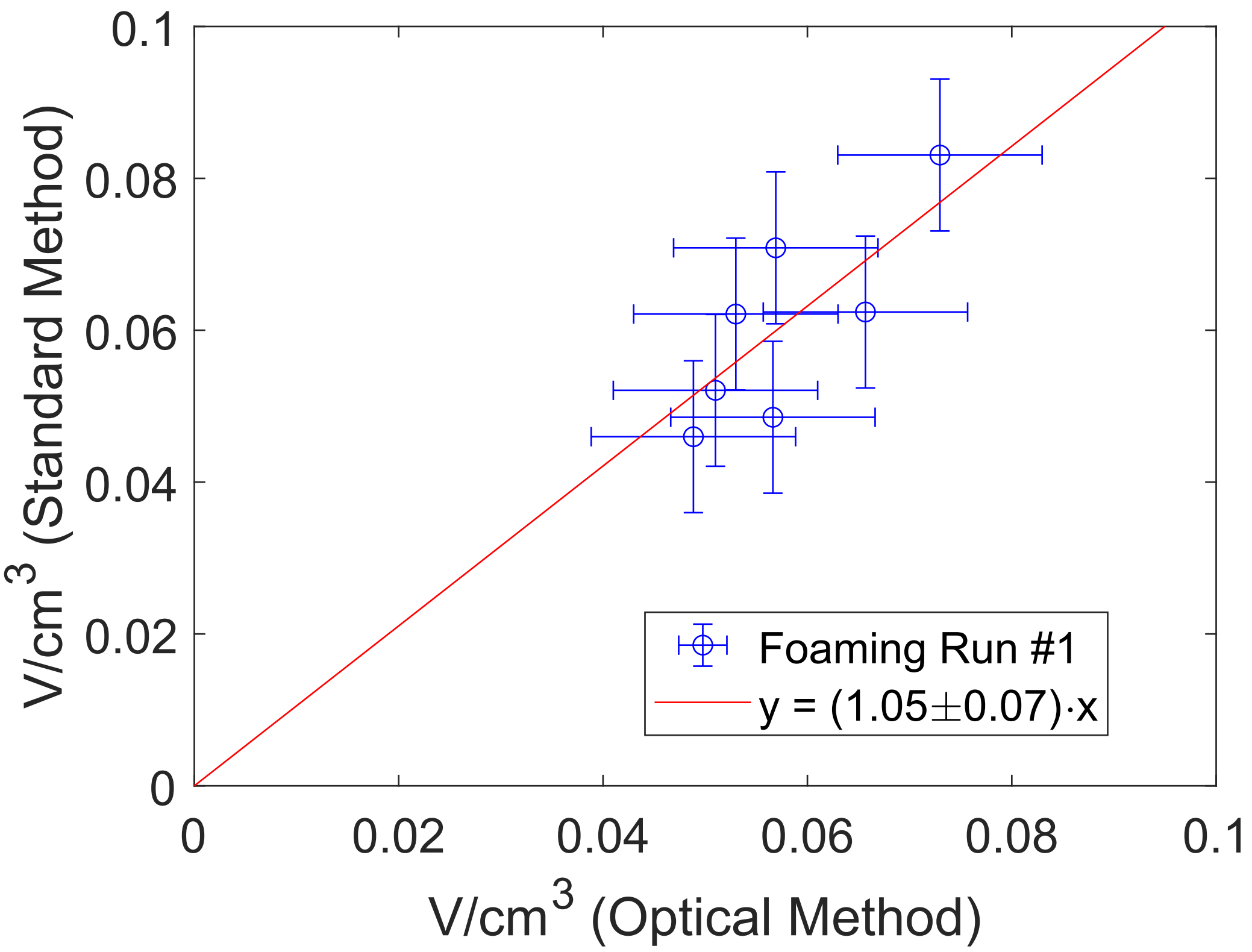

Figure S3. Comparison between the volume measurement conducted with the newly proposed optical method and the standard method based on liquid water

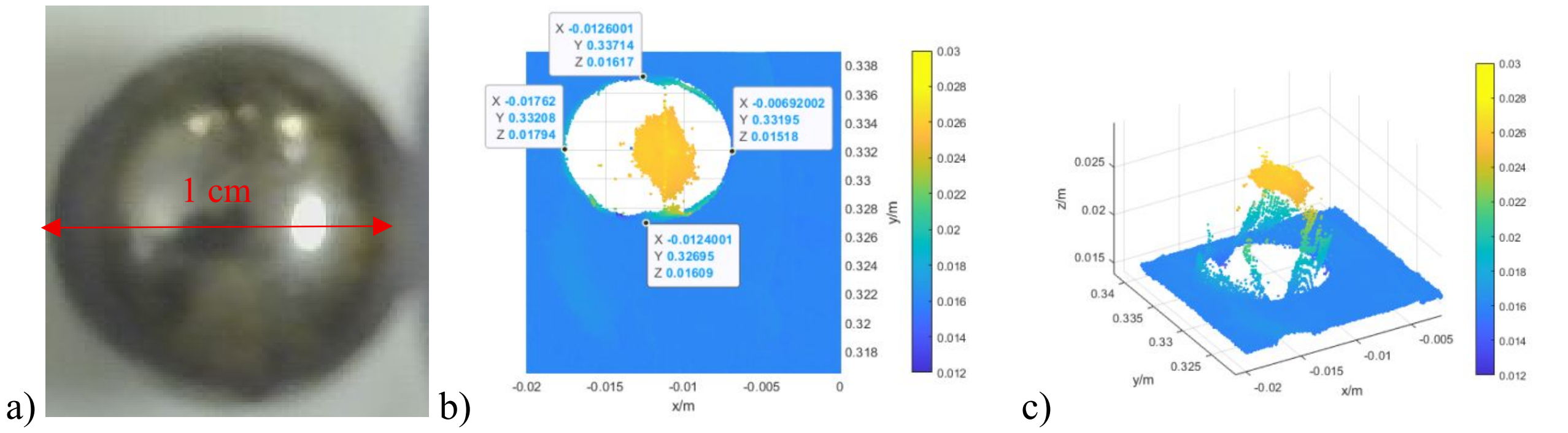

Figure S4. Stainless-steel bearing with polished surface placed on a flat Teflon sheet substrate. The diameter of the sphere is equal to 1 cm. a: picture of the bearing. b: top view of the scanned height profile. c: 3D plot of the measured height profile

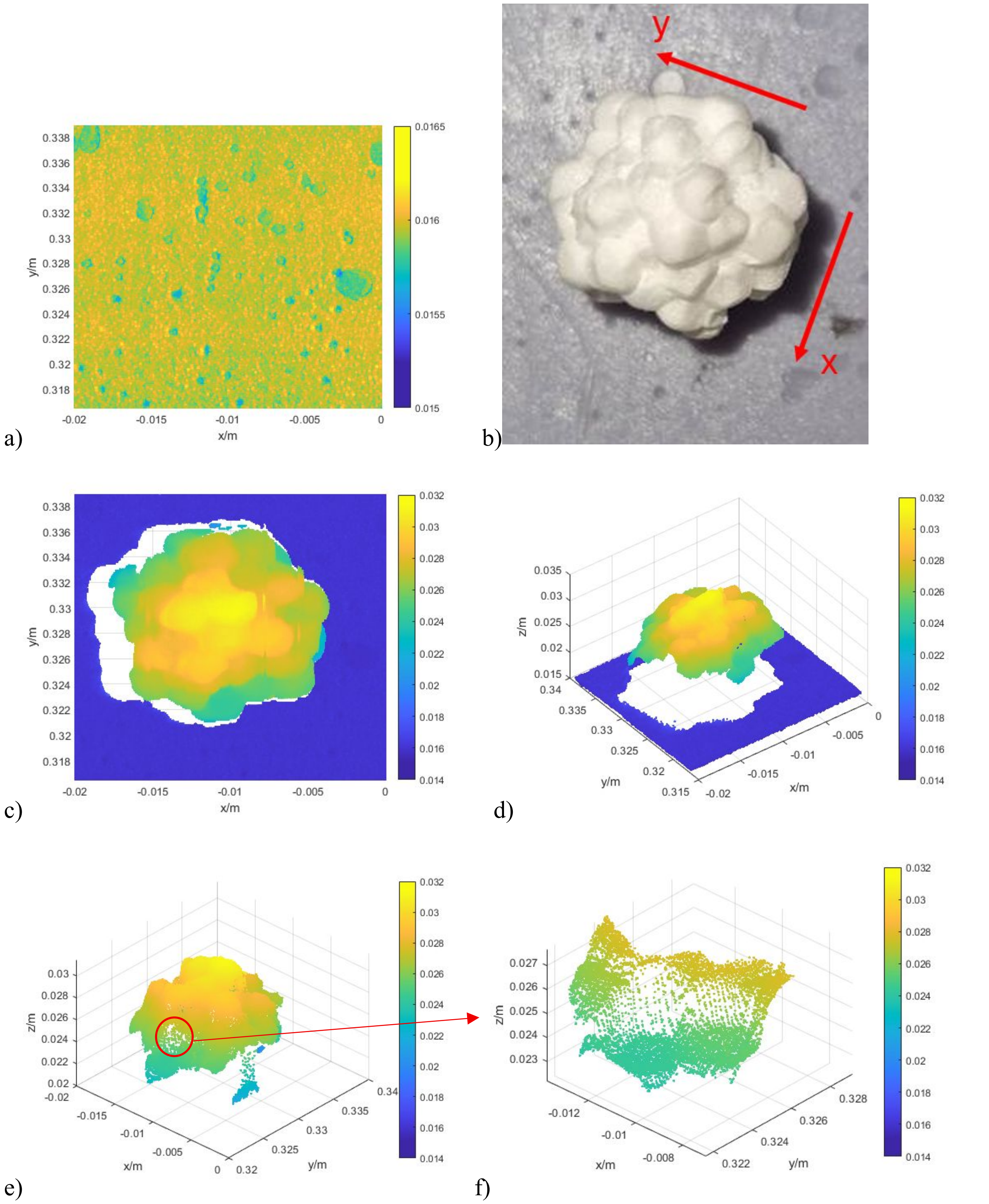

Figure S5. a: surface of the plate where the PS sample is placed for the profile measurement. b: picture of the PS foam analyzed with the scanner profilometer. Axes directions are reported for comparison with figures c to f. c: top view of the scanned foam. d: zoom plot. f: zoom plot corresponding to red circle in d highlighting missing points in the height profile.

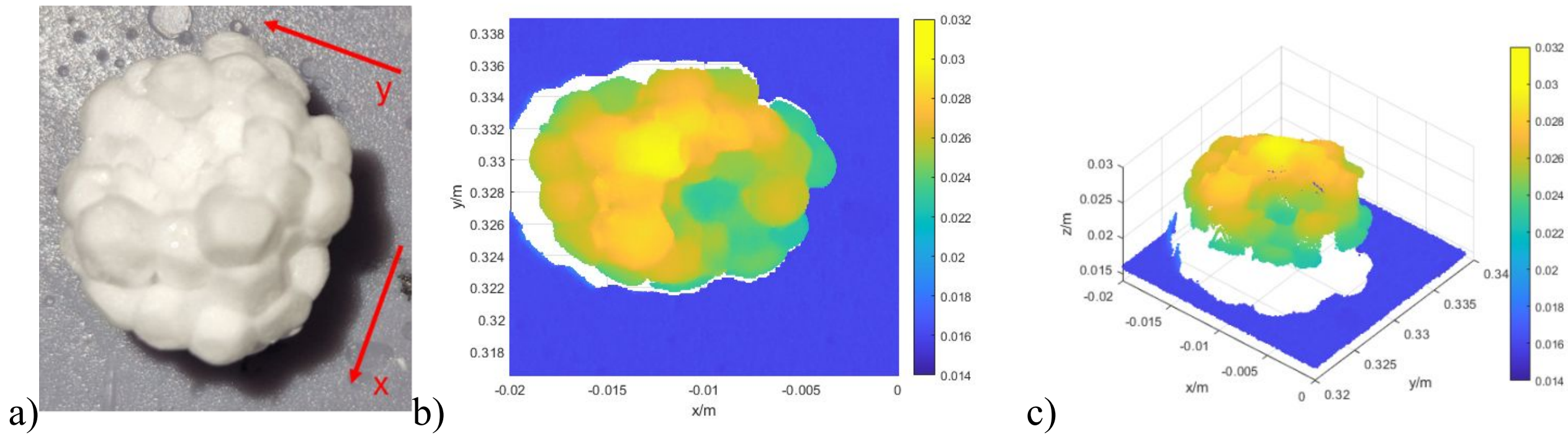

Figure S6. Commercial polystyrene foams for heavy-duty cushioning in packaging applications. a: picture of the tested foam. Axes directions helps the reader compare the picture with the scanned height profile. b: top view. c: 3D profile scan

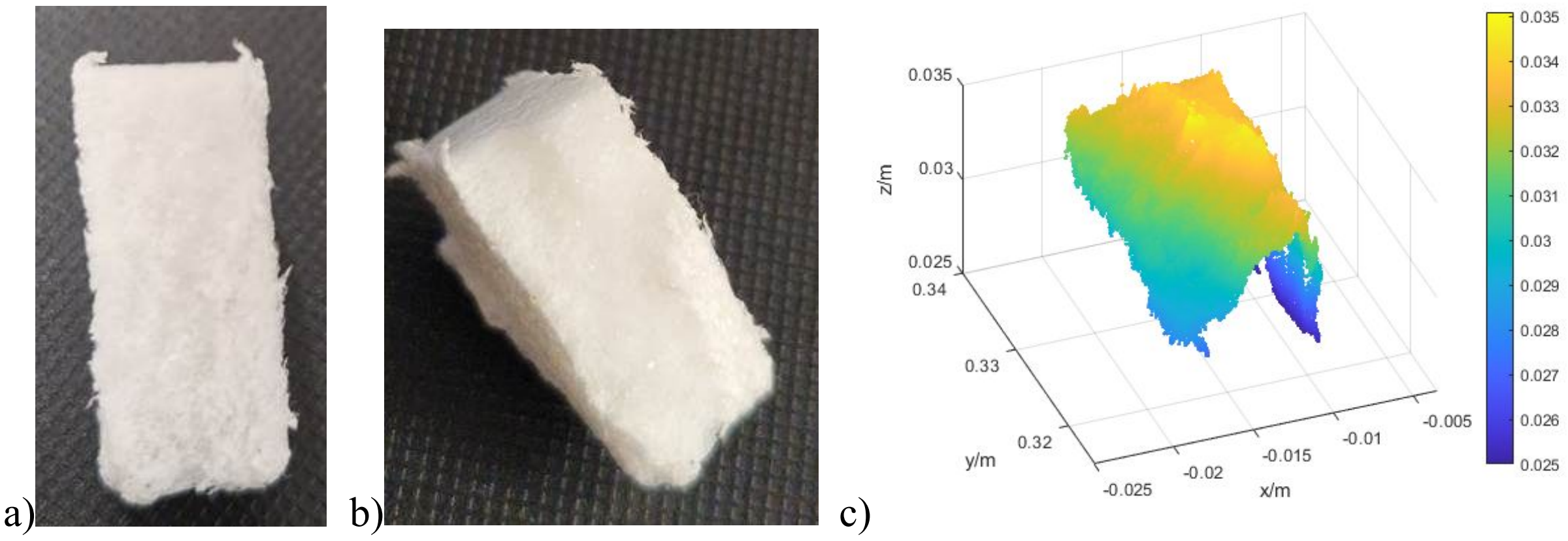

Figure S7. PET foam. a: top view of the scanned surface. b: side view of the scanned surface. c: scanned profile.

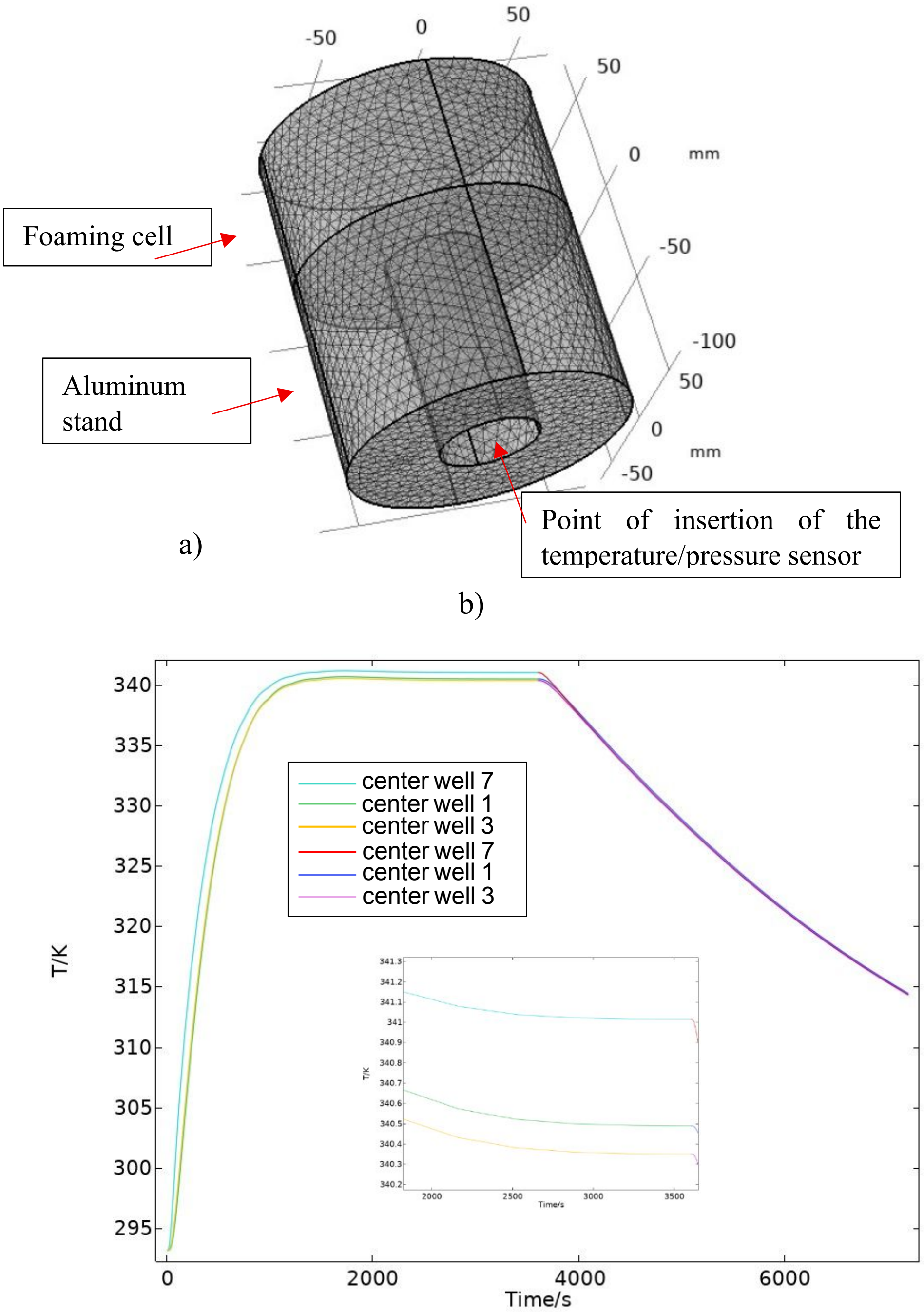

Figure S8. Theoretical description of the temperature profiles in the system. A: mesh grid and geometry of the foaming cell made of stainless steel and the aluminum stand. B: temperature profiles at the center of the wells number 1, 3 and 7. The inset shows temperature differences at steady state during heating.
